# Blindness in Sudan: Is It Time to Scrutinise Survey Methods?

**DOI:** 10.1371/journal.pmed.0030476

**Published:** 2006-12-19

**Authors:** Hannah Kuper, Clare Gilbert

## Abstract

Kuper and Gilbert discuss the limitations of a new survey of blindness in a war-torn area in southern Sudan, published in PLoS Medicine.

Those who have been involved in surveys, particularly in developing countries, will agree that doing a survey is a challenging undertaking. Conducting surveys entails explaining the purpose and procedures in understandable language, walking around villages through rain or sun, and coaxing resistant people into being examined. This must all be done while adhering to strict research protocols, carrying out the enumeration meticulously and the examinations with patience and care. Surveys are all the more difficult in low-income settings where experienced staff and sampling frames for enumeration may not be available. We therefore applaud the efforts of Ngondi and colleagues in carrying out a survey of blindness in a war-torn area in southern Sudan, which is published in *PLoS Medicine* [1].

Estimates from the survey suggest that the prevalence of blindness in people over five years old was 4.1%, which is far higher than previously reported for Africa [2]. The prevalence also vastly exceeds the World Health Organization estimate, based on data from across the continent, that 1% of people in Africa are blind [3]. Should the results of this survey be a cause for utter despair, or should we instead scrutinise the survey methodology?

## A Comparison with Other Surveys

Without doubt the conditions in this area in southern Sudan are dire, and the survey had to use the personnel available. However, surveys conducted in other severely underserved or postconflict areas in Africa show a far lower prevalence of blindness. For instance, a survey conducted in 1998 in a rural area of the Democratic Republic of Congo estimated that only 0.5% of surveyed people over 10 years old were blind in both eyes [4].

**Random Walk**

The random walk method is a technique for sampling households in a door to door survey. Starting at a central place in the village, a random direction is chosen (e.g., by spinning a bottle). A household is chosen at random among those along the line from the centre to the edge of the village. The next closest household is visited, in turn, until the cluster has been completed.

Perhaps the high prevalence found by Ngondi and colleagues is because the people in southern Sudan are particularly vulnerable to eye disease? The leading cause of blindness in their survey was cataract (which occurs all over the world), responsible for 41% of cases, followed by trachoma (an infectious disease found mainly in Africa), causing 35% of cases. A recent survey in Ethiopia in an area hyperendemic for trachoma found a similar proportion of blindness due to trachoma (23%), but with a far lower prevalence of blindness overall (8% in people aged over 40 years compared to 23% in the over 50s in southern Sudan) [5]. Even areas endemic for onchocerciasis (another infectious cause of blindness) in Central African Republic, where almost three quarters of cases of blindness are due to onchocerciasis, report about half the prevalence of blindness observed in the southern Sudan survey [6].

Aside from the high prevalence of blindness, the distribution of blindness within the population of southern Sudan was also unusual. Among the people enumerated aged over 50 years it was rare to find someone without visual impairment, as only 17% of women and 32% of men aged over 50 years had normal vision in both eyes. Moreover, the typical ratio of blindness to low vision for Africa is about 1:3 [3], whereas in the survey in southern Sudan a ratio of 1:2 was reported. Populations usually experience about twice as much unilateral blindness as bilateral blindness, but in the southern Sudan survey the number of people blind in both eyes was approximately the same as those blind in one eye only.

## Sources of Bias

Taking these pieces of evidence together it seems likely that in Ngondi and colleagues' survey blind people were over-sampled, or blindness was over-diagnosed, or the normally sighted were no longer living in the area. There are two essential steps to conducting a survey. The first is the selection of participants and the second is determining whether or not they have the disease of interest, in this case blindness. Biases can occur at either of these steps.

The integrated eye care workers (IECWs) who carried out the survey had previously worked in an eye surgery camp, where the whole purpose is to find as many blind people as possible. Surveys require a totally different approach, and it may have been difficult for the IECWs not to include blind people who had been denied eye care for so very long. Alternatively the bias may have occurred through the use of the random walk methodology (see sidebar), as the authors acknowledge. The random walk method allows an element of subjectivity in the selection of households for the survey. Blindness is almost unique among diseases as it can be diagnosed by people in the village (one does not have to be a health professional to suspect blindness). The village guides accompanying the teams know that the teams are looking for blind people and they also know where these blind people live. This makes it difficult to stop “helpful” village guides from steering enumeration teams towards blind people.

The other type of bias can occur during the eye examination. Measuring visual acuity is difficult and needs the right conditions, such as sufficient light, lack of glare, and few distractions. The IECWs may not have been highly experienced at measuring visual acuity, and it is not clear how much they were supervised in the field. Errors in measuring visual acuity occur when normally sighted people misread the charts (by accident or through poor conditions) and are labelled as visually impaired. In contrast, blind people cannot achieve good vision and read the charts correctly. The bias is, therefore, only in the direction of overestimation.

## Interpreting the New Survey

This leaves us with two possible interpretations. The first is that the prevalence of blindness in this survey has been over-estimated, perhaps because enumeration or visual acuity measurements were not undertaken to the standards required. The second is that these prevalence estimates are accurate, and reflect large-scale out-migration from the area (including death) of those with normal vision.

Putting aside the methodological flaws in the survey, the prevalence of blindness is outside the range previously reported, and the pattern of eye disease observed is highly unusual. It is, of course, likely that blindness is a serious problem in southern Sudan and another survey to confirm the very high prevalence is indicated.

## Abbreviations

IECW: integrated eye care worker
